# Supplementary material for: Enhanced Vasculogenic Capacity Induced by 5-Fluorouracil Chemoresistance in a Gastric Cancer Cell Line
Source: Int J Mol Sci. 2021 Jul 19;22(14):7698. doi: 10.3390/ijms22147698 (PMC8303918; doi:10.3390/ijms22147698)
Supplement: Supplementary file 1 [file ijms-22-07698-s001.zip › Table S1.pdf]

node1	node2	node1 accession	node2 accession	coexpression score
EPHA2	LGALS3	ENSP00000351209	ENSP00000254301	0.065
KDR	PECAM1	ENSP00000263923	ENSP00000457421	0.062
KDR	TYMP	ENSP00000263923	ENSP00000379038	0.049
LGALS3	EPHA2	ENSP00000254301	ENSP00000351209	0.065
PECAM1	KDR	ENSP00000457421	ENSP00000263923	0.062
PECAM1	TYMP	ENSP00000457421	ENSP00000379038	0.091
TYMP	KDR	ENSP00000379038	ENSP00000263923	0.049
TYMP	PECAM1	ENSP00000379038	ENSP00000457421	0.091
