# Supplementary figures and images for: Enhanced Vasculogenic Capacity Induced by 5-Fluorouracil Chemoresistance in a Gastric Cancer Cell Line
Source: Int J Mol Sci. 2021 Jul 19;22(14):7698. doi: 10.3390/ijms22147698 (PMC8303918; doi:10.3390/ijms22147698)

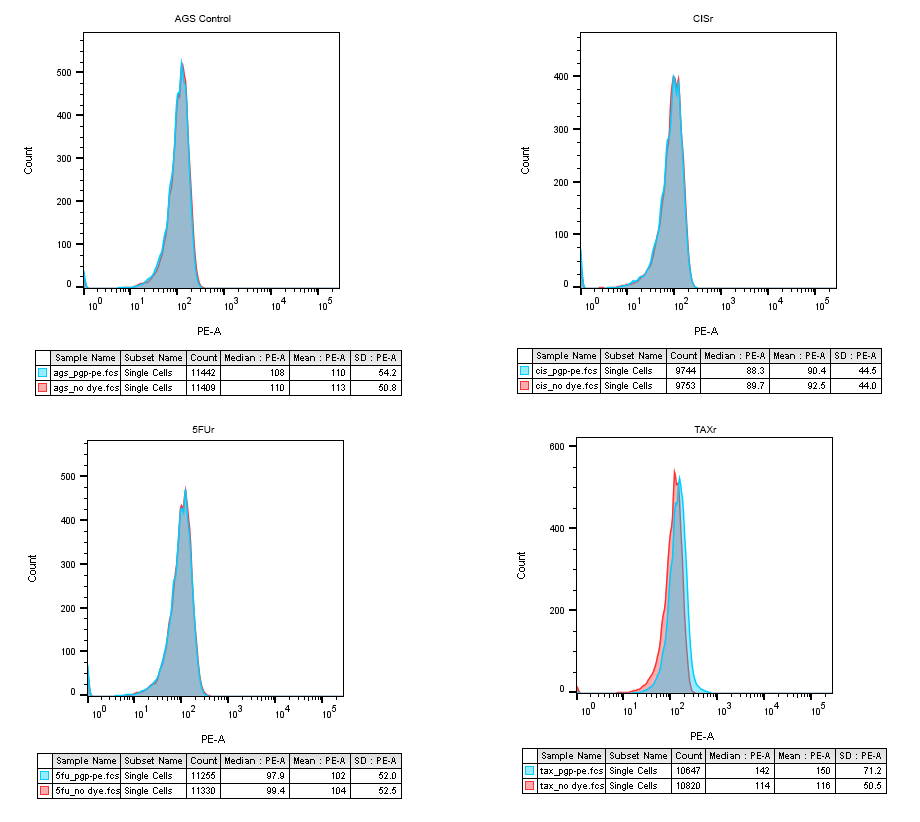

Supplement: Supplementary file 1 [file ijms-22-07698-s001.zip › Figure S1.tif]

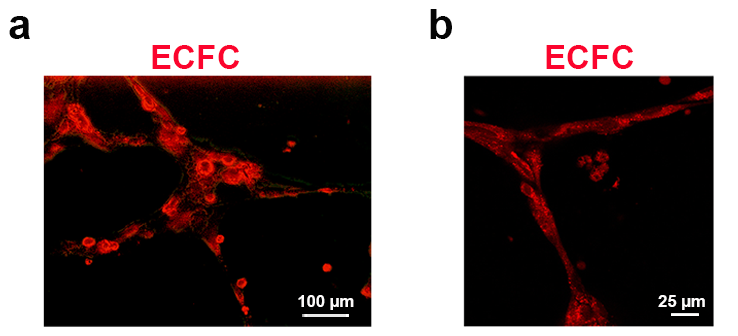

Supplement: Supplementary file 1 [file ijms-22-07698-s001.zip › Figure S2 mod.tif]

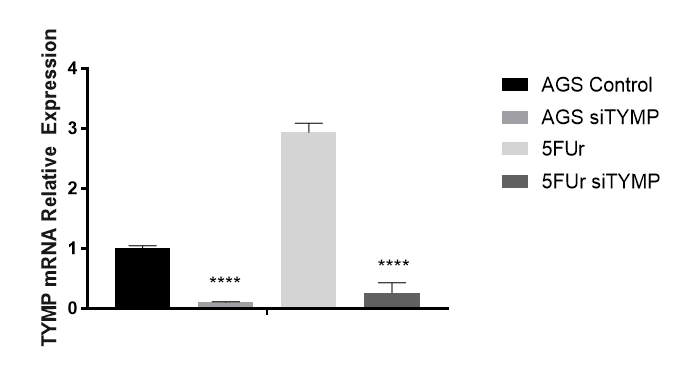

Supplement: Supplementary file 1 [file ijms-22-07698-s001.zip › Figure S3.tif]

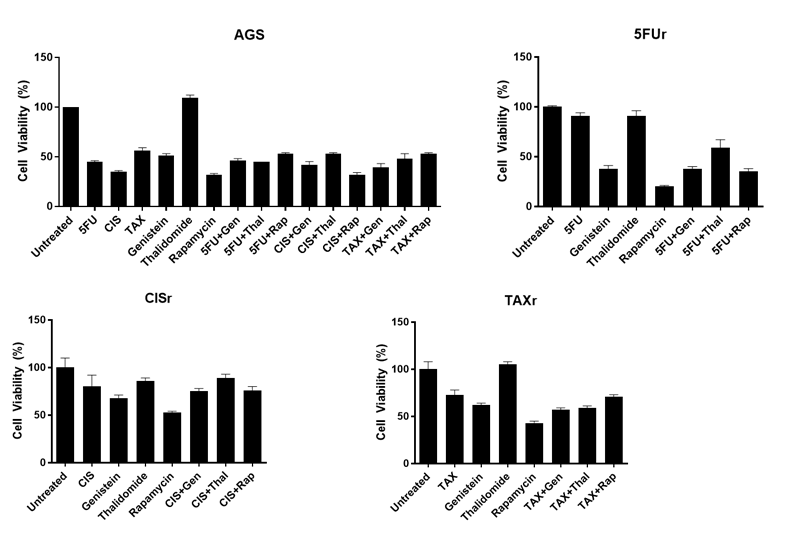

Supplement: Supplementary file 1 [file ijms-22-07698-s001.zip › Figure S4.tif]
